# Case of Subacute Poliomyelitis Anterior, with Commencing Phthisis

**Published:** 1884-09

**Authors:** R. Shingleton Smith

**Affiliations:** Physician to the Bristol Royal Infirmary, Honorary Fellow of King's College, London


### CASE OF SUBACUTE POLIOMYELITIS ANTERIOR, WITH COMMENCING PHTHISIS. Treatment by Ergotin and Iodoform. Recovery. By R. SHINGLETON SMITH, M.D., M.R.C.P., Physician to the Bristol Royal Infirmary, Honorary Fellow of King's College, London.

Thomas B., æt. 23, bootmaker, married eighteen months, one child, was admitted to the Bristol Royal Infirmary on March 20th, 1884, with partial paralysis of both arms. Had always been strong and steady since thirteen years old, when he was laid up a month with fever. Had been getting thin for last twelve months, but was in good health till one month ago, when, after being in wet clothes for three hours, he began to have pain in the lower part of bowels, and afterwards shooting down both legs to his feet; this pain was very severe, and kept him awake night after night. Three weeks ago he found he could not pick up rivets in left hand; there was no pain in the hands, but tingling in the tips of the fingers. No history of injury to back or shock of any kind could be elicited. Has been in the habit of drinking beer freely, sometimes all the day twice in a week, but never had

*delirium tremens;* for the last six months has not had more than one pint a day. There is no evidence, nor is there a history, of syphilis. He has always had complete control over the bladder and bowels. For a week past not able to have complete coitus, although usually has done so about six times a week. Has been unable to walk upstairs for one week, from want of power to lift up legs.

There is no history of paralysis in family; but one brother died of consumption at eleven, and another brother is now ill with "consumption of bowels." Father and mother and seven brothers and sisters are healthy.

*Present condition.*—Thin, high cheek-bones, cheeks fallen in, teeth good; not anæmic; body fairly muscular, bones large. Is able to walk, and stands with eyes closed; is rather laboured and shaky in walking, but does not drag either leg; fell down on attempting to walk upstairs. Is able to lift his legs up from the bed, but only with considerable effort; arms are wasted, and he has very imperfect muscular action of hands. Is not able to cut up his food, or dress himself; can just unfasten his shirt-buttons. Is not able to lift up either arm to the horizontal line; grasp is feeble with both hands. Unable to abduct arm from side; on arm being lifted up, it falls again immediately. Pronation of fore-arm perfect; supination impaired; flexion of hand impaired; extension almost absent. Small opposition of thumb to forefinger. Thenar muscles much wasted; fingers somewhat claw-like, from wasting of interossei. Deltoids are absolutely powerless, but not wasted. Sensation is normal everywhere. Reflexes, plantar, triceps, tendo-achillis, and knee-jerk absent; no ankle clonus. Cremasteric reflex present. Sight good; pupils normal. Pulse 84. Electrical reactions: fore-arms, front and back, respond to the normal induced current; but thenar muscles are sensibly diminished in irritability, especially the right. The leg muscles respond normally.

The facial muscles appear to be wasted, but there is no impairment of their action; he can whistle, blow out cheeks, open and screw up lids, laugh, &c.

The breathing is chiefly abdominal, but the thoracic movement is perfect.

Fibrillary tremors are constantly visible in the fore arms, arms, and shoulder muscles.

The patient was ordered to remain at rest in the recumbent position, was to have a liberal diet, to take two grains of ergotin in pill every four hours, and on

March 29th, oleate of mercury, 10 per cent., was applied down the spine twice daily.

March 28th. Electrical reactions fairly good, except in thenar and hypothenar eminences, which contract only feebly with a strong current; wasting of those muscles very marked.

March 29th. Deltoids quite paralysed, but not markedly wasted. Constant fibrillary tremors of deltoids, rhomboids, infra-spinati, and other scapular muscles; slight diminution of faradic reaction in scapular muscles; rhomboids, latissimus dorsi, and lumbar muscles respond well to normal strength. The leg muscles respond well, and are not now wasting.

The ergotin was increased to four grains every four hours.

April 4th. Arms not gaining strength; grasp is very deficient. Patient has a difficulty in raising himself from recumbent posture; shuffles with difficulty on to left elbow, and then pushes himself up with a jerk. He complains of twitchings in the legs, and constant muscular tremors are visible in the extensors of fore arms, as well as in the muscles about the shoulders. There is no dysphagia, and speech is normal. No complaint of pains in limbs or back. Occasional headache, in consequence of which the ergotin was reduced to two-grain doses.

April 5th. Patient feels weak, has no pain, but marked twitchings of all the muscles of limbs. Left thumb muscles give only slight response to strongest secondary current of one-celled Stöhrer battery; right thumb muscles give none.

Liq. Vesicatorius was applied on each side of the spine, at middle and lower cervical regions, for about six inches in length.

April 11th. He feels stronger, has no pains and no sickness, not now complaining of headache. Is again taking four grains of ergotin every four hours.

April 22nd. In consequence of diarrhœa, the ergotin was discontinued.

April 26th. Muscular power much improved. Though he cannot cut up his dinner, yet he can feed himself. The grasp of both hands is much stronger, although still weak. Is now able to raise the left arm above his head, but not the right.

General health has not been satisfactory. He complains of cough, has a little expectoration, and there is a patch of dulness, with feeble breath-sounds, and crepitation below the left clavicle. Temperature has been of a hectic type *(see over)*.

Sputum examined for tuberculous bacilli, but with a negative result. Ordered iodoform, one grain three times a day in pill, and cod-liver oil, a teaspoonful thrice daily.

May 3rd. Grasp had greatly improved; can now do up his shirt-buttons, and can raise both arms above his head rapidly and without difficulty. Electric reactions now almost normal; thumb muscles respond to a current only slightly stronger than normal. Lung sounds much improved. No dulness or crepitation at the left apex, less cough, no expectoration. Taking two grains of iodoform three times a day. Made out-patient May 9th; steadily improving.

| | | M. | E. | | | M. | E. |
|---|---|---|---|---|---|---|---|
| March | 20th. | 98·6 | 100 | April | 12th. | 99·2 | 99·6 |
| ,, | 21st. | 98·4 | 99·4 | ,, | 13th. | 99 | 101·4 |
| ,, | 22nd. | 98·4 | 100 | ,, | 14th. | 98·2 | 101 |
| ,, | 23rd. | 99 | 100·4 | ,, | 15th. | 98·6 | 100·6 |
| ,, | 24th. | 99·4 | 100 | ,, | 16th. | 98 | 100·6 |
| ,, | 25th. | 99 | 99·2 | ,, | 17th. | 98·6 | 100 |
| ,, | 26th. | 98·6 | 100 | ,, | 18th. | 98·4 | 100·6 |
| ,, | 27th. | 99·6 | 100·6 | ,, | 19th. | 98·4 | 101 |
| ,, | 28th. | 99·6 | 100·4 | ,, | 20th. | 98·8 | 101 |
| ,, | 29th. | 98·8 | 100·4 | ,, | 21st. | 98·6 | 102 |
| ,, | 30th. | 99·4 | 100 | ,, | 22nd. | 100 | 101 |
| ,, | 31st. | 99 | 101·2 | ,, | 23rd. | 98·8 | 100·2 |
| April | 1st. | 101 | 101·4 | ,, | 24th. | 98·8 | 102 |
| ,, | 2nd. | 100·4 | 101·4 | ,, | 25th. | 98·8 | 99·6 |
| ,, | 3rd. | 99·4 | 100·8 | ,, | 26th. | 98·6 | 99 |
| ,, | 4th. | 99 | 100·6 | ,, | 27th. | 98·6 | 100 |
| ,, | 5th. | 98·6 | 100·6 | ,, | 28th. | 98·6 | 101 |
| ,, | 6th. | 99 | 102 | ,, | 29th. | 99 | 99 |
| ,, | 7th. | 99 | 101·6 | ,, | 30th. | 98·6 | 99·4 |
| ,, | 8th. | 99·2 | 101·4 | May | 1st. | 98·4 | 99 |
| ,, | 9th. | 98·4 | 100 | ,, | 2nd. | 98·4 | 99 |
| ,, | 10th. | 99 | 101 | ,, | 3rd. | 98·4 | 98·6 |
| ,, | 11th. | 99 | 101·4 | ,, | 4th. | 98·4 | 98·6 |

This case presents several points of interest, of which the principal are the following:—

The rapidly-increasing loss of power was manifest from day to day, and the diminution in electric reaction was decided from week to week; there appeared also to be muscular wasting in the fore-arms and hands, but this could not be remarked in the measurements taken at different times.

There could be no room for doubt as to the diagnosis. The disease was too acute for progressive muscular atrophy, and the diminution of electric contractility where there was not much muscular wasting excluded this hypothesis. The usual indications of myelitis and meningitis were altogether absent, but the existence of an evening pyrexia gave rise to the idea that there might be a more active inflammatory process in the cord than is usual with poliomyelitis. A perusal of the record of temperature will shew that the variations observed from time to time were not dependent on the disease of the cord which gave rise to the muscular weakness and wasting, inasmuch as the temperature was not much above the normal at the time when the weakness was steadily increasing, whereas at a later period, when the evening temperature was 101·4° to 102°, the muscular condition was either stationary or improving. Careful observation of the daily temperature led to the suspicion that some other than the spinal cord disease was giving rise to the evening hectic; and then the existence of cough, with expectoration and suspicious physical signs of disease at the apex of the left lung, gave an explanation of the anomalous temperature such as would not be likely to result from a subacute or chronic poliomyelitis.

As regards the lung disease, there was no evidence of anything wrong at the time of the patient's admission, but afterwards the dulness, with defective breathing and moist sounds, gave clear evidence of mischief in the left apex. What was the nature of this disease? The examination of the sputum gave only negative evidence; no tuberculous bacilli could be found on one occasion, and afterwards the expectoration had so rapidly subsided under the influence of iodoform, that none could be obtained for subsequent examination. The character of the pyrexia, the general aspect and symptoms of the patient, his family history, and his physical signs, all appeared to bear out the view that there was a patch of tubercular disease at the apex of the lung. On the other hand, the rapidity with which the dulness cleared up, and the cough and expectoration subsided, support the negative view given by the one examination of the sputum, that the disease in the lung may have been of a simple inflammatory character.

The case appears to shew the value of ergotin in the treatment of early spinal disease; it also gives evidence in favour of iodoform in early lung disease.

P.S.—On May 27th the patient again came under observation; his muscular power had steadily improved, and he had gained in weight five pounds in three weeks. But the lung condition was not satisfactory; cough had been troublesome for ten days, and there was much pain round the left side of his chest. Loud rasping friction sound was audible from the left clavicle down to the fourth rib, and there was infra-clavicular dulness. Pulse was 120, temp. 99°.

